# Association between self-care agency and depression and anxiety in patients with diabetic retinopathy

**DOI:** 10.1186/s12886-021-01883-w

**Published:** 2021-03-06

**Authors:** Bo Zhang, Qin Wang, Xuancan Zhang, Li Jiang, Lezhi Li, Bangshan Liu

**Affiliations:** 1 Xiangya School of Nursing, Central South University, 172 Tongzipo Road, Changsha, 410013 Hunan China; 2 Department of Ophthalmology, The Second Xiangya Hospital, Central South University, Changsha, 410011 Hunan China; 3 Department of Nursing, The Second Xiangya Hospital, Central South University, Changsha, 410011 Hunan China; 4 Department of Psychiatry, The Second Xiangya Hospital, Central South University, Changsha, 410011 Hunan China; 5 Mental Health Institute of Central South University, China National Clinical Research Center on Mental Disorders (Xiangya), China National Technology Institute on Mental Disorders, Hunan Technology Institute of Psychiatry, Hunan Key Laboratory of Psychiatry and Mental Health, Changsha, 410011 Hunan China

**Keywords:** Depression, Anxiety, Self-care agency, Diabetic retinopathy

## Abstract

**Background:**

This study aimed at investigating: 1) the relationship between self-care agency and depression and anxiety; 2) the sociodemographic and clinical factors associated with depression and anxiety in patients with diabetic retinopathy (DR) in China.

**Methods:**

A cross-sectional study was conducted. One-hundred and five patients with DR were recruited. Self-care agency was assessed by the exercise of self-care agency (ESCA) scale. Depression and anxiety were assessed by the hospital anxiety and depression scale (HADS-D and HADS-A). Pearson or Spearman correlations were performed to assess the association between self-care agency and depression and anxiety. Stepwise multivariate linear regression analyses were conducted to assess the contribution of the sociodemographic and clinical factors to depression and anxiety.

**Results:**

Thirty-six (34.3%) and 43 (41.1%) patients exhibited depressive and anxiety symptoms, respectively. Only 24 (22.9%) patients showed a high self-care agency. The ESCA total and subscale scores were negatively correlated depressive symptoms (*P* < 0.05). Self-care skills were negatively correlated with anxiety symptoms (P < 0.05). ESCA total score, rural residence, history of hypertension and visual acuity were associated with depression; self-care skills and diastolic blood pressure were associated with anxiety.

**Conclusions:**

Self-care agency negatively correlates with depression and anxiety in patients with DR. Special attention should be paid to patients living in rural areas and/or those having a history of hypertension when assessing depression and anxiety in patients with DR. Future studies are needed to clarify the causal relationship between self-care agency and depression and anxiety.

## Background

Diabetes mellitus (DM) is a heterogeneous group of physiological dysfunctions characterized by hyperglycemia, insulin resistance, insufficient insulin secretion, or glucagon hypersecretion [1]. Diabetic retinopathy (DR) is a common microvascular complication of DM. DR is the main cause of preventable blindness and visual impairment (VI) in working aged people [2]. It has been estimated that one third of people with DM in the world have symptoms of DR, and about one in ten of them develop vision-threatening DR. [3] Thus, dealing with DR is an urgent need for the global health system.

Previous studies have shown that DM is associated with increased risk of depression [4] and anxiety [5], and complications of DM, such as DR, further increase the risks [6, 7]. Rees et al. reported that 15.4 and 22.7% of patients with DR exhibit symptoms of depression and anxiety, respectively [6]. Elevated depression and anxiety could not only bring higher disease burden for patients with DR, but also impede proactive glucose control and result in accelerated progression of DR. [8] Thus, special attention should be paid to the risk factors and effective interventive strategies for depression and anxiety in patient with DR.

Due to the chronicity of DM, self-care is of key significance in its management. The competence of self-care is usually assessed by self-care agency. Self-care agency, according to the definition by Orem [9], is one’s learned capability to engage in self-care behaviors. It has been reported that good self-care agency predicts good glycemic control and adherence to medications, well-preserved quality of life and low rate of complications, such as DR and diabetic neuropathy [10, 11]. Self-care agency is also essential for the prevention and management of depression [12]. Previous studies have shown that self-care agency is negatively correlated with risk of depression in community-dwelling elders [13, 14]. However, currently no study has investigated the association between self-care agency and depression and anxiety in patients with DR.

In this study, we aimed to assess the correlation between self-care agency and depression and anxiety in patients with DR for the first time. We hypothesized that low self-care agency would be associated with higher levels of depression and anxiety in patients with DR. We further aimed to investigate the sociodemographic and clinical factors associated with depression and anxiety in patients with DR.

## Methods

### Participants

One-hundred and five patients with DR were recruited from the inpatient department of Ophthalmology of the Second Xiangya Hospital from October 2019 to January 2020 through a convenience sampling procedure. The inclusion criteria for the participants were: 1) met the diagnostic criteria of diabetic Retinopathy of the Chinese Guideline for the clinical management of diabetic retinopathy (2014) [15]; 2) age ≥ 18 years; 3) signed written informed consent to participate the study. The exclusion criteria for the participants were: 1) patients with severe systemic organic diseases; 2) patients with retinopathy due to trauma and other reasons; 3) patients with severe mental disorders, communicative or cognitive impairment. This study was approved by the ethics committee of the Second Xiangya Hospital of Central South University on July 8th, 2019. The number of Institutional Review Boards approval was S301. Written informed consent was obtained from all participants in this study. The participants were not involved in the design of the study.

### Assessments

#### Sociodemographic and clinical information

Sociodemographic and clinical information were obtained by a self-designed questionnaire. The sociodemographic information includes age, gender, education, marriage status, residence (rural or urban). The clinical information includes duration of DM, duration of VI, blood pressure, hemoglobin A1c (HbA1c) at admission, history of smoking, history of hypertension, family history of DM. The clinical information, including HbA1c and visual acuity were collected within three days of the assessment of depression, anxiety and self-care agency. Visual acuity was measured by the standard logarithmic visual acuity chart. “VI” was defined as the presenting vision acuity of the better eye. We used this definition because it reflects real-life vision well. Levels of VI was classified as blindness, severe VI, moderate VI, mild VI and no VI based on the criteria of the International Classification of Diseases 11 classification system [16]. The number of participants classified as blindness (LogMAR ≥1.30), severe VI (1 ≤ LogMAR<1.30), moderate VI (0.48 ≤ LogMAR<1), mild VI (0.30 ≤ LogMAR<0.48) and no VI (LogMAR<0.30) of the worse eye were 25, 8, 27, 10 and 35, respectively. Due to the limited number of participants with severe VI or mild VI, we grouped the visual acuity into “moderate or severer VI” and “mild or no VI” for further analyses. Sixty patients were categorized as “moderate or severer VI” and 45 patients categorized as “mild or no VI” with the cut-off of 6/18 (LogMAR = 0.48).

#### Self-care agency

Self-care agency was assessed by the most commonly used scale in this domain: Exercise of Self-Care Agency (ESCA). The ESCA scale was developed by American scholars Kearney et al. [17], based on Orem’s self-care theory. It was translated into Chinese by Taiwan scholars Wang et al. and exhibited good reliability and validity in a sample of Taiwan women [18]. The scale includes 43 items, comprising four subscales: self-concept, self-nursing responsibility, self-care skills and health knowledge level. The self-concept scale assesses the participant’s cognition about his/her behavioral pattern or personality traits. One typical item of self-concept is “I know my strong and weak points”. The self-nursing responsibility subscale assesses one’s understanding about his/her responsibility in keeping healthy and/or preventing the development of the disease. One example item of self-nursing responsibility is “I take responsibility for my own actions”. The self-care skills subscale assesses one’s acquired skills about keeping healthy and addressing the influence of the disease. One typical item of self-care skills is “I eat a balanced diet”. The health knowledge subscale assesses one’s knowledge about his health and disease. One example item of health knowledge is “I understand my body and how it functions”. Each item has a 5-point Likert scale, ranging from 0 (very uncharacteristic of me) to 4 (very characteristic of me). Eleven items (item 3, 6, 10, 16, 19, 22, 25, 28, 32, 34 and 39) were scored reversely. The total score of ESCA ranges from 0 to 172, with higher total and subscale scores indicating stronger self-care agency. Subjects with scores < 33.3% (≤56), 33.3–66.6% (57–114) and > 66.6% (≥115) of the maximum score are considered with low, moderate and high self-care agency, respectively. The Cronbach’s α coefficient of the ESCA scale in this study was 0.84.

#### Depression and anxiety

Depression and anxiety were assessed by the Hospital Anxiety and Depression Scale (HADS). The HADS scale includes 14 items, with 7 items assessing anxiety and 7 items assessing depression, comprising the anxiety (HADS-A) and depression (HADS-D) subscales. Each item has a 4-point Likert scale, ranging from 0 (no, definitely not) to 3 (yes, definitely). Six items (item 2, 4, 6, 7, 10 and 14) are scored reversely. The total score is the sum of each item scores, with higher total score indicating more severe anxiety or depression symptoms. HADS has been proven to be an effective tool for assessment of anxiety and depression either in developed or developing countries, with good reliability and validity [19]. In this study, the Cronbach’s α coefficient of the HADS scale was 0.88. A cut-off score of 9 was recommended for HADS-D and HADS-A in signifying the clinical meaningful depression and anxiety in Chinese population, respectively [20].

### Data collection

Data were collected on an online survey platform (www.wjx.cn) using an ipad. Since the patients had VI, the scales were filled with the help of the authors in a face-to-face manner. The authors read and explained the items to the patients, and recorded the patients’ answers. Standardized instructions and explanations for the items were developed to improve consistency between the two authors.

### Statistical analyses

Statistical analyses were conducted in SPSS 25.0 software for Windows. Kolmogorov-Smirnov test (*n* > 50) and visual inspection of histogram were used to test the normality of the continuous variables. Continuous data were described by the mean and standardized deviation (SD) for variables with normal distribution and by the median and interquartile range (IQR) for variables with skewed distribution. Count data were described by number of cases and percentages. Differences between groups were explored by using t test or Chi-square test. Pearson or Spearman correlations were performed to assess the association between the sociodemographic, clinical variables and depression and anxiety, respectively. Finally, stepwise multivariate linear regression analyses were conducted to assess the relative contribution of the variables showing significance in the univariate analysis to the variance of depression and anxiety. Statistical significance was set at a two-tailed, α = 0.05.

## Results

### Characteristics of the sociodemographic and clinical information

The sociodemographic and clinical information of the included participants are shown in Table 1. The sample consisted of 51 (48.6%) males and 54 (51.4%) females, aged 28–86 (56.7 ± 11.6) years. Sixty-seven (63.8%) patients had an education level of junior high school or below (≤ 9 years). Most (92.4%) of the patients were married. About half (47.6%) the participants lived in urban areas and the other half (52.4%) lived in the rural areas.

**Table 1:** Sociodemographic and clinical characteristics of the participants

Variable	N (%) ***N*** = 105
**Categorical variables**	
Gender	
Female	54 (51.4)
Male	51 (48.6)
Education	
< 9 y	67 (63.8)
≥ 9 y	38 (36.2)
Marital status	
Married	97 (92.4)
Others	8 (7.6)
Residence	
Urban	50 (47.6)
Rural	55 (52.4)
History of smoking	
Y	40 (38.1)
N	65 (61.9)
History of hypertension	
Y	71 (67.6)
N	34 (32.4)
Family history of DM	
Y	47 (44.8)
N	58 (55.2)
Level of VI^*^	
Blindness	25 (23.8)
Severe VI	8 (7.6)
Moderate VI	27 (25.7)
Mild VI	10 (9.5)
No VI	35 (33.3)
**Continuous variables**	
Age, m (sd), y	56.7 (11.6)
HbA1c, m (IQR), %	7.4 (6.5–8.6)
BP, m (sd), mmHg	
Systolic	142.0 (17.3)
Diastolic	82.8 (9.8)
Duration of DM, m (IQR), y	10.0 (5.0–15.0)
Duration of VI, m (IQR), m	7.0 (2.0–15.0)
HADS, m (sd)	14.9 (6.9)
HADS-A, m (sd)	7.74 (3.82)
HADS-D, m (sd)	7.14 (3.84)
ESCA, m (sd)	103.1 (13.7)
Self-concept, m (IQR)	19.0 (17.0–21.5)
Self-nursing responsibility, m (IQR)	14.0 (12.0–16.0)
Self-care skills, m (IQR)	29.0 (26.0–31.0)
Health knowledge level, m (sd)	40.8 (6.8)

DM: diabetes mellitus; y: years; m (sd): mean (standardized deviation); m (IQR): median (interquartile range); HbA1c: hemoglobin A1c; BP: blood pressure; DM: diabetes mellitus; HADS-D: hospital anxiety and depression scale - depression; HADS-A: hospital anxiety and depression scale – anxiety; ESCA: the exercise of self-care agency scale. * classification of VI is based on the International Classification of Diseases 11 classification system

For the clinical information, 54 (51.4%) patients had a history of DM ≥ 10 years. Forty-four (41.9%) patients had VI for more than 12 months. All of the participants suffered from type 2 diabetes and were at the advanced stage of DR (proliferative DR). Forty (38.1%) patients had a smoking history and 47 (44.8%) had a DM family history. Seventy-one (67.6%) of the patients had comorbid hypertension.

### Self-care agency and depression and anxiety

The total score of the ESCA scale was 103.1 ± 13.7, with only 24 (22.9%) patients scoring high (≥115) and 81 (78.1%) patients scoring moderate. No patient scored low in the ESCA scale. The HADS-D score was 7.14 ± 3.84 and the HADS-A score was 7.74 ± 3.82, respectively. Thirty-six (34.3%) and 43 (41.1%) patients exhibited clinical meaningful symptoms of depression (HADS-D ≥ 9) and anxiety (HADS-A ≥ 9), respectively, among which 26 (24.8%) exhibited symptoms of both depression and anxiety.

### Association between the sociodemographic and clinical variables, self-care agency and depression and anxiety

Patients who lived in rural areas showed significantly higher level of depression (*P* < 0.01) and anxiety (*P* = 0.05) than those living in urban areas (Table 2). Patients with hypertension showed significantly higher level of depression (P < 0.01) but not anxiety than those with no hypertension (Table 2). Patients with blindness or severe impairment in visual acuity showed significantly higher level of depression (*P* = 0.02) but not anxiety than those with mild-to-moderate impairment (Table 2). The ESCA total score, self-concept, self-care skills and health knowledge level were negatively correlated depression symptoms (*P* < 0.01, P = 0.02, P < 0.01, respectively; Table 2). Self-care skills were negatively correlated with anxiety symptoms (P < 0.01; Table 2). Diastolic blood pressure (BP) was positively correlated with anxiety (*P* = 0.01). We found no relationship between age, gender, education, marital status, history of smoking, family history of DM, HbA1c level, duration of DM and duration of VI with depression and anxiety (Table 2).

**Table 2:** Association between the sociodemographic and clinical variables and depression and anxiety

Variable	Depression		Anxiety	
	Mean (SD)	P	Mean (SD)	P
Gender				
Female	7.2 (4.0)	0.95	8.4 (3.9)	0.08
Male	7.1 (3.7)		7.1 (3.6)	
Education				
< 9 y	7.5 (3.8)	0.24	8.1 (4.2)	0.16
≥ 9 y	6.6 (3.8)		7.1 (3.0)	
Marital status				
Married	7.1 (3.9)	0.79	7.9 (3.9)	0.25
Others	7.5 (2.8)		6.3 (3.0)	
Residence				
Urban	5.7 (3.3)	0.00^*^	6.9 (2.9)	0.02^*^
Rural	8.4 (3.9)		8.5 (4.4)	
History of smoking				
Y	7.3 (4.1)	0.74	7.2 (3.7)	0.28
N	7.1 (3.7)		8.1 (3.9)	
History of hypertension				
Y	7.9 (3.7)	0.00^*^	8.1 (3.5)	0.17
N	5.6 (3.7)		7.0 (4.3)	
Family history of DM				
Y	7.5 (3.7)	0.38	7.5 (3.7)	0.51
N	6.8 (3.9)		8.0 (3.9)	
VI				
Mild or no VI	5.2 (2.4)	0.04^*^	6.3 (2.2)	0.29
Moderate or severer VI	7.5 (3.6)		7.8 (3.9)	
**Continuous variables**				
Age	-0.14^a^	0.16	-0.01^a^	0.95
HbA1c	-0.02^b^	0.84	−0.07 ^b^	0.49
BP				
Systolic	0.00 ^a^	0.99	−0.18 ^a^	0.07
Diastolic	0.02 ^a^	0.87	0.24 ^a^	0.01^*^
Duration of DM	−0.05 ^b^	0.60	0.03 ^b^	0.75
Duration of VI	0.06 ^b^	0.56	−0.05 ^b^	0.59
ESCA total score	−0.31 ^a^	0.00^*^	−0.16 ^a^	0.11
Self-concept	−0.23 ^b^	0.02^*^	−0.11 ^b^	0.26
Self-nursing responsibility	−0.17 ^b^	0.09	−0.08 ^b^	0.42
Self-care skills	−0.40 ^b^	0.00^*^	−0.29 ^b^	0.00^*^
Health knowledge level	−0.28 ^a^	0.00^*^	−0.11 ^a^	0.27

^a^ Pearson correlation; ^b^ Spearman correlation; ^*^ *P* < 0.05; y: year; HbA1c: hemoglobin A1c; BP: blood pressure; DM: diabetes mellitus; HADS-D: hospital anxiety and depression scale - depression; HADS-A: hospital anxiety and depression scale – anxiety; ESCA: the exercise of self-care agency scale

The multiple linear analyses revealed that residence (β = − 1.94, 95% CI, − 3.29 - − 0.60), history of hypertension (β = − 1.91, 95% CI, − 3.34 - − 0.49), visual acuity (β = − 0.98, 95% CI, − 1.75 - − 0.21) and ESCA total score (β = − 0.57, 95% CI, − 0.11 - -0.01) were independently associated with depression (Table 3). For every reduction in ESCA total score, there was a 0.57 increase in HADS-D. Those who lived in urban areas were more likely to have lower levels of depression than those who lived in rural areas. Those who have no history of hypertension were more likely to have lower levels of depression than those who have history of hypertension. Those who have mild-to-moderate impairment in visual acuity were more likely to have lower levels of depression than those who are blind or have severe impairment in visual acuity. Self-care skills (β = − 0.24, 95% CI, − 0.43 - -0.06) and diastolic BP (β = 0.09, 95% CI, − 0.17 - -0.02) were independently associated with anxiety (Table 3). For every reduction in self-care skills, there was a 0.24 increase HADS-A. For every increase in diastolic BP, there was a 0.09 increase in HADS-A. Residence, history of hypertension, visual acuity and ESCA total score explained 25% of variance in depression. Self-care skills and diastolic BP explained 12% of variance in anxiety.

**Table 3:** Multiple linear regression analysis of association between the sociodemographic and clinical variables, self-care agency and depression and anxiety

Variables	β	P
**Depression** ^**a**^		
Residence		
Rural	0 [Reference]	
Urban	−1.94 (−3.29 - -0.60)	0.00
History of hypertension		
Y	0 [Reference]	
N	−1.91 (−3.34 - -0.49)	0.01
Visual acuity		
Moderate or severer VI	0 [Reference]	0.03
Mild or no VI	−0.89 (−1.63 - -0.13)	
ESCA total score	−0.57 (− 0.11 - -0.01)	0.03
**Anxiety** ^**b**^		
Self-care skills	−0.24 (− 0.43 - -0.06)	0.01
Diastolic BP	0.09 (−0.17 - -0.02)	0.01

^a^: variables selected from the following variables: residence, history of hypertension, ESCA, self-concept, health knowledge; ^b^: variables selected from the following variables: residence, DBP, self-care skills; R^2^ for the depression model: 0.23, *P* = 0.00; R^2^ for the anxiety model: 0.12, *P* = 0.00; β: regression coefficient; BP: blood pressure; HADS: hospital anxiety and depression scale; ESCA: the exercise of self-care agency scale

## Discussion

This study is the first to assess the relationship between self-care agency and depression and anxiety in patients with DR in China. Consistent with our hypothesis, we found negative correlations between self-care agency and depression and anxiety, with most ESCA subscales correlated with depression while only self-care skills correlated with anxiety. We also found that residence and history of hypertension are associated with depression and diastolic BP is associated with anxiety in patients with DR. Our findings stressed the association between self-care agency and depression, calling for attention to assessment of self-care agency when assessing psychological well-being in patients with DR.

Our study revealed a close relationship between self-care agency and depression, rather than anxiety. It is possible that anxiety exerts double-edged effects on the sufferers: on one side, individuals with anxiety are hypervigilant and would actively take actions to promote health and prevent illness, such as utilizing excessive health care service; on the other hand, these actions are often ineffective or unnecessary [21]. The double-edged effects may neutralize each other and result in scarce relationship between self-care agency and anxiety.

The negative relationship between self-care agency and depression is consistent with the previous findings in patients with DM [10]. It is best explained by mutual causality about the relationship between self-care agency and depression. On one hand, self-care may improve adherence to medications, facilitate regular and pleasant self-engaged activities and promote social interaction, which are all important to improve the quality of life and psychological well-being in patients with a chronic condition [22, 23], such as DR. Self-care activities may also help patients keep aware of their emotional status and seek help when they get distressed [24, 25]. On the other hand, depression may bring about negative thoughts about one’s self-care agency [26, 27]. Individuals with depression may struggle in taking effective actions to improve health or prevent illness, such as regular exercise, balanced diet and interpersonal communication [28]; they may also ignore their sources and advantages which can be utilized for self-care, illness curation and prevention [29]. Thus, self-care and psychological well-being may mutually influence each other and construct a vicious circle in the progression of diabetes and DR.

The circularity of self-care agency and psychological wellbeing suggests that interventions for one part of the circle would bring about changes to the other part. Pharmacological and psychological interventions for depression have been widely investigated. It should be stressed that the self-care agency is an acquired capability that can be improved by education [30, 31]. Thus, it is reasonable to deduce that educational interventions aiming at improving self-care agency will help attenuate depression in patients with DR, which is a promising direction and should be tested in future studies.

The finding of urban patients exhibiting lower levels of depression and anxiety than those living in rural areas is also important. There is an increasing gap in the economy development between the rural and urban areas in recent years [32]. Generally, urban areas provide better social welfare for disabled people than rural areas. Thus, the lower levels of depression and anxiety in urban patients may be associated with better economic status and social welfare for these patients. The findings of association between comorbid hypertension with depression and of diastolic BP with anxiety are also consistent with previous findings of interaction between emotion and blood pressure [33, 34], warranting active interventions in controlling hypertension in patients with DR.

Interestingly, we didn’t find a correlation between other sociodemographic (age, gender, education, marital status) and clinical (duration of DM, duration of VI, HbA1c) variables and depression and anxiety in our study, which is inconsistent with a previous study [6]. We believe it may be attributed to the difference in the characteristics of the sample in our study and that of Rees, et al. In Rees et al. [6], the authors included DM patients with or without VI and found that patients with severe VI and increased HbA1c are associated with higher level of depression. In our study, however, only patients with DR were included. Generally, DR is a complication occurring at the advanced stage of DM, indicating that patients with DR had lived with DM for a long time. In this case, patients may have developed adaptation to the emotional distress of DM, neutralizing the effect of DR duration and suboptimal glycemic control on depression and anxiety. However, future studies are needed to testify our hypothesis.

Our study has three strengths: first, we used well-validated scales to assess self-care agency and depression and anxiety, which added robustness to our results; second, we administered the scales in a face-to-face manner, which optimized our data quality; third, we collected the information on sociodemographic and clinical characteristics of the sample, which allowed us to adjust for potential confounders in our analysis. However, attention should be paid to several limitations of our study in the interpretation of our findings. First, this is a cross-sectional study with relatively small sample size. Future studies with larger sample size are needed to verify our findings. Second, the regression models in our study demonstrated that the identified variables could only explain a small proportion of variance in depression and anxiety, which means that some potentially important variables may have been omitted. For instance, the history of depression and anxiety and the severity of DR may exert significant influence on the current depressive and anxiety symptoms [6]. Future studies should take these variables into account and conduct a comprehensive assessment of the potential influencing variables. Third, Rasch analysis was not conducted on the data which may mean measurement precision was reduced; however, given that the ESCA and HADS are well-validated scales with good psychometric properties, we believe that the results of our study will nonetheless be robust.

## Conclusions

Our study firstly investigated the relationship between self-care agency and depression and anxiety in patients with DR. We found a negative correlation between self-care agency and depression and anxiety in patients with DR, suggesting a role of improving self-care agency for alleviating depression and anxiety. We also found that living in rural areas and comorbid with hypertension are associated with increased depressive symptoms, calling for special attention for patients with these characteristics.

## Data Availability

The datasets used and/or analyzed during the current study are available from the corresponding author on reasonable request.

## Abbreviations

DR: diabetic retinopathy
DM: Diabetes Mellitus
VI: visual impairment
ESCA: exercise of self-care agency
HADS: hospital anxiety and depression scale
HbA1c: hemoglobin A1c
BP: blood pressure
SBP: systolic blood pressure
DBP: diastolic blood pressure
